# Allicin prevents H_2_O_2_-induced apoptosis of HUVECs by inhibiting an oxidative stress pathway

**DOI:** 10.1186/1472-6882-14-321

**Published:** 2014-08-30

**Authors:** Sisi Chen, Yuye Tang, Ying Qian, Ruyi Chen, Lin Zhang, Like Wo, Hui Chai

**Affiliations:** College of Life Science, Zhejiang Chinese Medical University, Hangzhou, 310053 China; Hangzhou pharmavaxin co., LTD, Hangzhou, 310052 China; The First Affiliated Hospital of Zhejiang Chinese Medicine University, Hangzhou, 310006 China

**Keywords:** Allicin, Human umbilical vein endothelial cell (HUVEC), Anti-apoptosis, H_2_O_2_

## Abstract

**Background:**

Allicin, a primary ingredient of garlic, has been proposed to possess cardioprotective properties, which are commonly mediated by improved endothelial function.

**Methods:**

To investigate the effect and mechanism of allicin on the apoptosis of human umbilical vein endothelial cells (HUVECs), we used Propidium iodide (PI) staining and Annexin V/ PI staining assays to establish a model of oxidative stress apoptosis induced by H_2_O_2_. MTT, RT-PCR and western-blot assays were used to detect the effects and mechanism of allicin on the model.

**Results:**

PI staining, Annexin V/ PI staining assays and morphological assessment suggest that the cell death induced by 0.5 mM H_2_O_2_ is primarily apoptotic. Conversely, allicin reverses the effect of H_2_O_2_ on cell death, suggesting a role in protecting HUVECs from apoptosis. We demonstrated that H_2_O_2_ activates PARP cleavage, reduces pro-Caspase-3 levels and activates Bax expression; however, allicin inhibits each of these apoptotic signaling indicators. Allicin also reduces the levels of malondialdehyde and increases the levels of superoxide dismutase, nitric oxide release and endothelial nitric oxide synthase mRNA, but has no significant effect on inducible nitric oxide synthase mRNA levels.

**Conclusion:**

These results demonstrate that allicin has powerful effects in protecting HUVECs from apoptosis and suggest that protection occurs via a mechanism involving the protection from H_2_O_2_-mediated oxidative stress.

## Background

Cardiovascular diseases (CVDs) are a category of chronic noncommunicable diseases causing high global mortality and have been a heavy social burden in many countries [1, 2]. Atherosclerosis - a progressive disease characterized by the accumulation of lipids and fibrous elements in the large arteries - constitutes the single most important contributor to this growing burden of cardiovascular disease [3]. Endothelial dysfunction is considered an early indicator of atherosclerosis, preceding angiographic or ultrasonic evidence of atherosclerotic plaques [4]. In addition to managing anabolism and exchange of blood and tissue fluids, endothelial cells also act as an endocrine gland. Endothelial cells produce and secrete multiple biologically active substances that help to maintain normal angiostasis and balance of blood. Considerable evidence indicates that oxidized low-density lipoprotein (ox-LDL) can cause the apoptosis of vascular endothelial cells through multiple pathways [5]. However the production process of ox-LDL is complicated, and it is stable for only 1 month at 4°C. Conversely, H_2_O_2_ is economical, simple and practical, so it is commonly used in injury models to replicate the effects of ox-LDL [6, 7].

Natural antioxidants are important for the prevention and treatment of atherosclerosis. Garlic has been studied extensively for its cardioprotective properties with very promising results [8]. Its primary active ingredient, 2-propene-1-sulfinothioic acid S-2-propenyl ester (also known as allicin), has been shown to alter the composition of fatty acids in mice or rats fed a high fatty acid diet [9, 10]. The aim of present study was to use H_2_O_2_ instead of ox-LDL to establish a model of oxidative stress and apoptosis in which to observe the intervention effect of allicin on endothelial cell apoptosis. The characterization of a new antioxidant drug may be beneficial as a novel strategy for the treatment of atherosclerosis.

## Methods

### Cell culture

HUVECs were obtained from Cambrex (Shanghai Biological Technology Co., Ltd., China) and were grown in Dulbecco's modified eagle medium (DMEM) supplemented with 10% heat-inactivated FBS (Hangzhou Sijiqing biological engineering materials Co., Ltd., China) at 37°C in a humidified atmosphere of 5% CO_2_. Cells were used at passage 4–6 for all experiments.

### Propidium iodide (PI) staining

HUVECs were cultured in 6 well plates (BD Falcon, USA) at a density of 2.0 × 10^5^ cells/well in DMEM supplemented with 10% FBS. One day after plating, the cells were washed and incubated in serum-free medium for 12 hours. The cells were then washed again and incubated with medium containing various concentrations of H_2_O_2_ (0.1, 0.5, 1.0 mM) for 12 hours. The cells were trypsinized, washed with PBS, and centrifuged at 1000 rpm/min for 5 min. The cells were then resuspended at a density of 1 × 10^6^ cells/ml, and the suspensions were fixed with 70% precooled ethanol at 4°C for 1 h. Next, the cells were centrifuged at 1000 rpm/min for 5 min, resuspended in 1 ml diluted PI (Shanghai Biological Technology Co., Ltd., China) and incubated in the dark at 4°C for 30 min. Flow cytometry was performed using a FACSCalibur (Backmancoulter, USA). Data were analyzed using CellQuest software (Becton–Dickinson). The amount of necrosis was determined as the percentage of PI-positive cells.

### Annexin-V/PI assay

Annexin-V/PI assays were performed using a commercial apoptosis assay kit (Roche, Switzerland) according to the manufacturer's instructions. Briefly, HUVECs were cultured in 6 well plates (BD Falcon, USA) at a density of 2.0 × 10^5^ cells/well and incubated in DMEM supplemented with 10% FBS. One day later, the cells were washed and incubated in serum-free medium for 12 hours. The cells were then washed again and incubated in medium with various concentrations of H_2_O_2_ (0.1, 0.5, 1.0 mM) for 12 hours. After incubation, the cells were trypsinized and washed with PBS. After centrifugation at 1000 rpm/min for 5 min, the cells were resuspended in 500 μL binding buffer at a concentration of 1 × 10^6^ cells/ml. The suspensions were transferred to 1.5-mL tubes, and 5 μL of Annexin V and 10 μL of PI solution were added. The cells were incubated in the dark at room temperature for 20 min, and flow cytometry was performed using a FACSCalibur (Beckmancoulter, USA). Data were analyzed using CellQuest software (Becton–Dickinson). The amount of apoptosis was determined as the percentage of annexin V-positive cells/PI-negative cells.

### MTT assay

As a measure of overall levels of cell death, HUVECs were assessed by 3-(4,5-dimethylthiazol-2-yl)-2,5-diphenyltetrazolium bromide (MTT) assay. HUVECs were plated onto 96-well plates and incubated in DMEM supplemented with 10% FBS. One day later, the cells were washed and incubated in serum-free medium for 12 hours. The cells were then were randomly divided into 6 groups: the normal control group (untreated cells), the model control group (H_2_O_2_ only), and the H_2_O_2_ plus allicin (98% purity, Shaanxi Ciyuan Biotech Co., Ltd, China) groups (1 μg/mL, 10 μg/mL, 20 μg/mL or 40 μg/mL allicin). These concentrations of allicin were selected to reflect a range of biological activities of the drug in HUVECs. Thirty minutes prior to the end of the incubation period, MTT was diluted 1:500 in 0.5% FBS DMEM culture medium and 200 μl was administered to each well. The plates were wrapped in aluminum foil to protect them from light and read using an enzyme-labeled instrument (Biotek ELX 800/FLX800).

### Western blot assay

For the extraction of proteins, cells were placed in RIPA Lysis Buffer (Beyotime Institute of Biotechnology, China) and centrifuged at 13000 rpm/min for 30 min at 4°C. Protein concentrations were assayed with a NanoDrop instrument, and 40 μg of protein from each sample were run on a 15% SDS-PAGE gels. The separated proteins were transferred onto PVDF membranes. After blocking with 5% nonfat dry milk in double-distilled water at room temperature for 1 h, membranes were washed 3 times with PBS containing 0.05% Tween (PBS-T) and incubated overnight at 4°C with primary mouse monoclonal antibody (anti-PARP, anti-pro-Caspase-3, anti-Bax or anti-β-actin) (Santa Cruz Biotechnology, USA) at a 1:500 dilution. The membranes were washed 3 times with PBS-T, followed by 1 h incubation at room temperature in a 1:5000 dilution of goat anti-mouse-IgG-HRP (Santa Cruz Biotechnology, USA). After incubation, membranes were washed 3 times in PBS-T. Antigen-antibody complexes were analyzed by ECL, and protein levels were quantified by densitometry. Data were normalized to the β-actin content of the same sample.

### Measurement of oxidative activity

The concentrations of malondialdehyde (MDA), sodium oxide dismutase (SOD) and nitric oxide (NO) were assessed using dedicated kits (Nanjing Jiancheng Biological Engineering Institute, China) according to the manufacturer’s protocols.

### Reverse transcription PCR (RT-PCR) assay

Total cellular RNA was extracted from HUVECs by the Trizol method (Bio Basic Inc., Canada). PCR amplification was performed in a 20 μL reaction volume. The primer sequences were as follows: eNOS forward, 5’-CCAGCTAGCCAAAGTCACCAT-3’, eNOS reverse, 5’-GTCTCGGAGCCATACAGGATT-3’; iNOS forward, 5’-AGCGGTAACAAAGGAGATAG-3’, iNOS reverse, 5’-CCCGAAACCACTCGTATT-3’; GAPDH forward, 5’-GTCATCCATGACAACTTTGG-3’, GAPDH reverse, 5’-GAGCTTGACAAAGTGGTCGT-3’. After an initial denaturation at 95°C for 5 min, the PCR conditions were as follows: 35 cycles of denaturation at 95°C for 30 s, annealing at 55°C for 30 s, and extension at 72°C for 30s. The PCR products were electrophoresed on a 1% agarose, and stained with ethidium bromide solution.

### Real-time quantitative PCR assay

Levels of endothelial nitric oxide (eNOS) mRNA expression were determined by real-time quantitative PCR. Triplicate reactions were run in a volume of 20 μL, containing 2 μL cDNA, 10 μL 2 × SYBR Green mix, 6 μL ddH_2_O, 1 μL PCR forward primer (10 μM), and 1 μL PCR reverse primer (10 μM). After an initial denaturation at 95°C for 5 min, the PCR conditions were as follows: 35 cycles of denaturation at 95°C for 30 s, annealing at 55°C for 30 s, and extension at 72°C for 30s.

The ΔΔCt (threshold cycle) method was used to calculate eNOS mRNA expression levels for each sample, with GAPDH as the reference gene.

### Statistical analysis

All data are expressed as mean ± SEM. Statistical analysis was performed using the Student’s t-test and ANOVA. Significance was accepted at *p* <0.05.

## Results

### H_2_O_2_ promotes apoptotic cell death of HUVECs

To characterize the effects of H_2_O_2_ in inducing cell death of HUVECs, we assessed morphological changes 12 h after exposure to a range of doses of H_2_O_2_ (0.1 mM, 0.5 mM and 1.0 mM). H_2_O_2_ promoted clear morphological changes to the cells, including cell shrinkage, karyopyknosis, and irregular nuclei. These results suggest that H_2_O_2_ induces programmed cell death in HUVECs.

To determine whether the effects of H_2_O_2_ on HUVEC cell death also may be explained in part by an increase in necrosis, we assessed the percentage of cells that were positive by PI staining. H_2_O_2_ caused an increase in PI positivity, which was most dramatic at the highest dose (Table 1). These results suggest that H_2_O_2_-induced cell death of HUVECs is mediated through both apoptotic and non-apoptotic pathways.

**Table 1:** **The positive rate of PI of HUVEC cells in each group**

Group (n = 3)	Necrosis rate (%)
normal HUVECs	2.5 ± 1.7
0.1 mmol/L H_2_O_2_	7.9 ± 1.0*
0.5 mmol/L H_2_O_2_	8.1 ± 2.1*
1.0 mmol/L H_2_O_2_	25.7 ± 2.5**

Values are presented as mean ± SD; **p < 0.01, *p < 0.05 compared with normal HUVECs.

**Figure 1:**
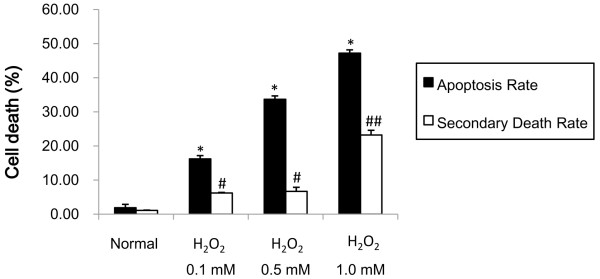
**Comparison of mortality rate and secondary death rate of HUVEC cells in each group.** The levels of apoptosis (apoptosis rate) and necrosis (secondary death rate) were determined by Annexin-V/PI assays 12 h after exposure to H_2_O_2_ at the indicated doses. Values represent the percentage of cells undergoing each form of death and are presented as mean ± SD; ^##^ *p* < 0.01, *p < 0.05, ^#^ *p* < 0.05 compared with normal HUVECs.

To further verify the effect of H_2_O_2_ in inducing cell death we performed AnnexinV/PI staining. This assay provides a measurement of both the apoptosis rate and a secondary death rate, which reflects the extent of necrotic cell death. Our results showed that both the apoptosis rate and secondary death rate were increased by H_2_O_2_, and that the increases were dose-dependent (Figure 1). However, that apoptosis rate rose more rapidly than the secondary death rate at lower H_2_O_2_ doses.

On the basis of the data in the PI staining and Annexin V/PI staining assays, we selected 0.5 mM H_2_O_2_ as a model dose that primarily causes apoptosis over necrosis for subsequent studies of apoptotic cell death.

### Allicin inhibits H_2_O_2_-induced HUVEC cell death

**Figure 2:**
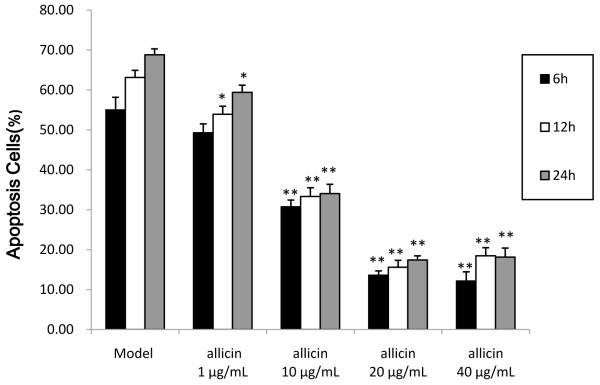
**Effects of allicin on cell death of HUVEC cells induced by 0.5 mM H**_**2**_ **O**_**2**_ **.** HUVECs were cultured with 0.5 mM H_2_O_2_ and various concentrations of allicin (1, 10, 20, 40 μg/mL) for 6, 12, or 24 hours. Thirty minutes prior to the end of the incubation period, MTT assays were performed to quantify metabolic activity. Each bar represents the mean ± SEM. *p <0.05, **p <0.01 compared with normal HUVECs.

To determine the effect of allicin on H_2_O_2_-induced apoptosis of HUVECs, we treated HUVECS with 0.5 mM H_2_O_2_ and a range of doses of allicin for 6, 12, or 24 h and then assessed cell death by MTT assay. While H_2_O_2_ promoted cell death in a time-dependent manner, allicin significantly reversed this effect (Figure 2). Because the dose of H_2_O_2_ selected for this experiment primarily causes apoptosis, these findings suggests that allicin may block an apoptotic pathway.

### Allicin inhibits the activation of an apoptotic cell death pathway by H_2_O_2_

**Figure 3:**
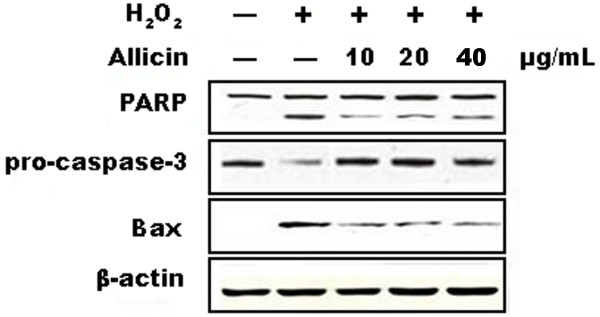
**Effects of allicin on the level of PARP, pro-Caspase-3 and Bax.** Levels of PARP, pro-Caspase-3 and Bax proteins were determined by Western blotting 24 h after exposure of HUVECs to 0.5 mM H_2_O_2_ and/or allicin as indicated. Membranes were probed with anti-β-actin antibody to verify equivalent sample loading.

Classic apoptotic cell death is enacted through a pathway that involves the cleavage of PARP and pro-Caspase-3 and the activation of Bax [11–13]. To determine whether H_2_O_2_ activates this pathway and whether allicin blocks apoptotic signaling, we assessed the levels of these proteins by Western blotting. HUVECs were treated with 0.5 mM H_2_O_2_ and a range of doses of allicin (10, 20, 40 μg/mL) for 24 h prior to analysis. Our results showed that H_2_O_2_ induced the cleavage of PARP, a decrease in pro-caspase-3 levels, and the activation of Bax expression; conversely, allicin inhibited these effects (Figure 3). These results further verify that 0.5 mM H_2_O_2_ activates an apoptotic pathway and that allicin inhibits the H_2_O_2_–mediated apoptosis.

### Allicin decreases oxidative activity in HUVECS by H_2_O_2_

**Figure 4:**
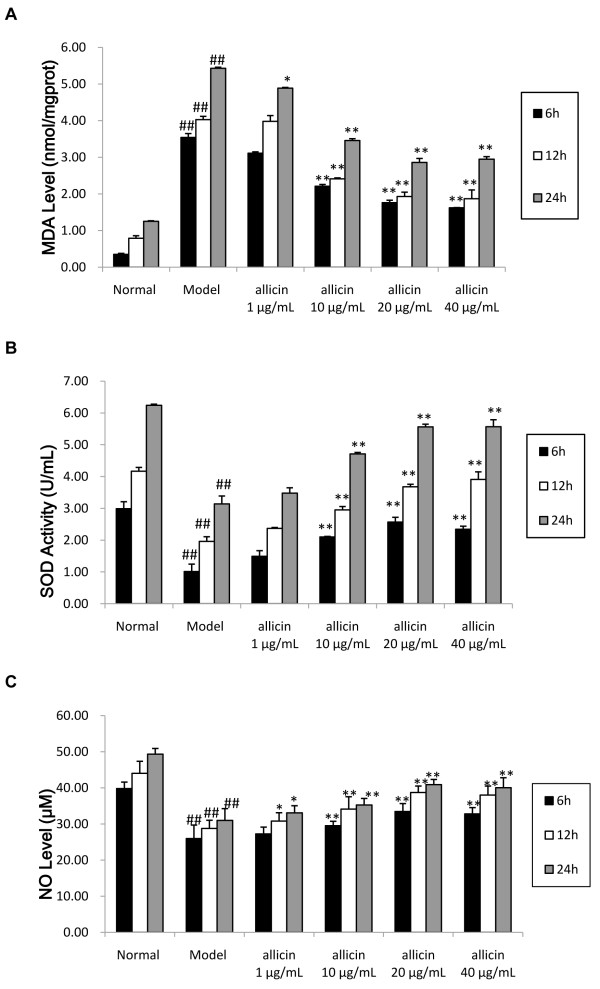
**Effect of allicin on oxidative activity in HUVECs induced by H**_**2**_ **O**_**2**_ **.** Levels of **(A)** the oxidative biomarker MDA, **(B)** the oxidative enzyme SOD, and **(C)** the oxidative signaling molecule NO were assessed in untreated cells (Normal), and cells treated with H_2_O_2_ only (Model) or H_2_O_2_ and allicin (allicin 10 μg/mL, 20 μg/mL and 40 μg/mL) for 6, 12, or 24 hours. Values represent the mean ± SE. ^##^ *p* <0.01 compared with normal HUVECs; *p < 0.05, **p < 0.01, compared with model group.

MDA is a biomarker of oxidative stress [14]. To determine whether allicin functions at the level of oxidative stress, we measured MDA levels in HUVECs following treatment with 0.5 mM H_2_O_2_ and allicin (1, 10, 20, 40 μg/mL) for 6, 12, or 24 hours. Our results showed that H_2_O_2_ causes a dramatic increase in MDA levels, which was reversed by allicin in a dose-dependent manner at all time points (Figure 4A).

To determine whether the effects on oxidative stress may be mediated by SOD, an enzyme that regulates oxidative stress [15], we measured SOD levels in HUVECs following H_2_O_2_ and allicin exposure. H_2_O_2_ significant decreased in SOD levels, and these levels were increased by concomitant allicin exposure (Figure 4B).

The effects of allicin on oxidative activity were further verified by assessing levels of NO, a free radical signaling mediator [16]. NO levels were significantly decreased in H_2_O_2_-induced HUVECs, and this decrease was reversed in a dose-dependent manner by allicin (Figure 4C).

**Figure 5:**
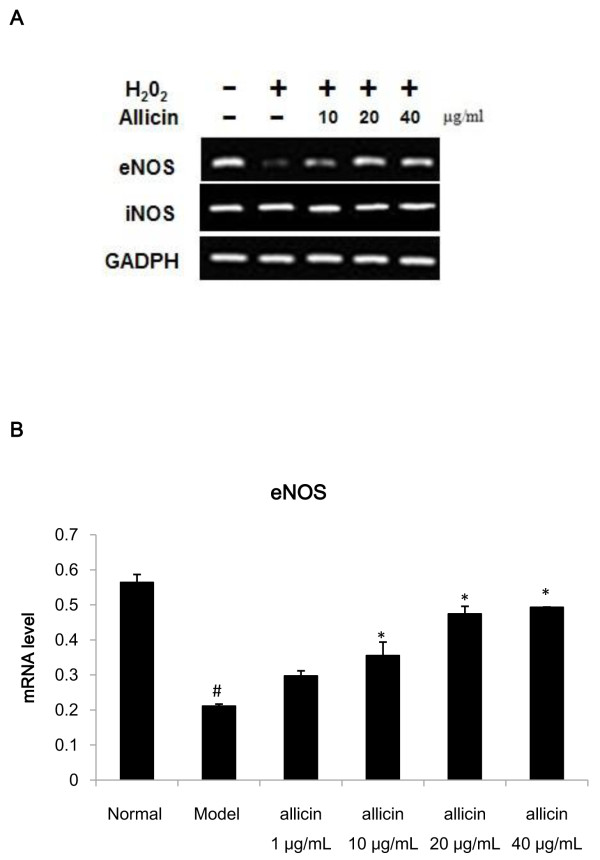
**Expression of eNOS and iNOS mRNA in HUVECs following H**_**2**_ **O**_**2**_ **and allicin treatment. (A)** Levels of eNOS and iNOS mRNA were determined by reverse transcription PCR following 12 h treatment with H_2_O_2_ and/or allicin as indicated. GADPH was used as an internal control. Each PCR product (5 μL) was separated on a 1.0% agarose gel. **(B)** Changes in eNOS mRNA expression were verified by real-time quantitative PCR. Values represent mean ± SD from three samples per group; ^#^ *p* < 0.05 compared with normal (untreated) HUVECs; *p < 0.05 compared with model (H_2_O_2_ only) group.

To further verify the effects of allicin on oxidative signaling, we measured levels of mRNAs for eNOS and iNOS, two enzymes that function in catalyzing the release of NO [17, 18]. eNOS mRNA expression was downregulated by H_2_O_2,_ and this downregulation was reversed by allicin; while iNOS mRNA expression remained unchanged (Figure 5). These results suggest that allicin may prevent H_2_O_2_-mediated apoptosis via the protection from detrimental oxidative activity mediated by eNOS production of NO and decreased SOD levels.

## Discussion

Apoptosis, a form of programmed cell death, is directly or indirectly regulated at the genetic level, as opposed to necrosis, which is based on extrinsic factors and for which the cell has no active role [19]. Apoptosis plays an important role in tissue remodeling, aging and response, and irreversible damage; and abnormal apoptosis may be the cause of many diseases.

Allium sativum (Liliaceae), whose common name is garlic, is an ancient spice and a medicine used for centuries around the world. Allicin (2-propene-1-sulfinothioic acid S-2-propenyl ester) is a key molecule of garlic and is responsible for the pungent smell of garlic [20]. A role for allicin has been widely demonstrated in cardiovascular prevention [21–27], but the specific role of allicin as the compound corresponding to this effect and its mechanisms have not been elucidated.

H_2_O_2_ has the same oxidation resistance as ox-LDL, is easier to produce, and is well established as a common model for oxidative injury [6, 7]. Consequently, we established a HUVEC oxidative stress model by using H_2_O_2_ instead of ox-LDL to induce HUVEC apoptosis. We determined the effect and mechanism of allicin on apoptosis of HUVECs induced by H_2_O_2_ at 0.1 - 0.5 mM. PI staining and Annexin-V/PI assay demonstrated that the apoptosis rate was increased, but an increase in the secondary mortality was not obvious. When the concentration of H_2_O_2_ was increased to 1 mM, the apoptosis rate was increased, but secondary mortality was also increased significantly. For this reason, we selected 0.5 mM H_2_O_2_ as an appropriate concentration for inducing optimal apoptosis, with minimal amounts of secondary necrosis.

MTT assay demonstrated that allicin effectively reduces the apoptosis of HUVECs induced by H_2_O_2_ in a dose-dependent manner. These results were verified by Western blotting, which suggests that allicin stabilizes pro-Caspase-3 protein expression and reduces PARP and Bax protein expression. Caspases are a well-characterized group of cysteine proteases, which are related in structure and reside in the cytosol. A common feature of caspases is the ability to disconnect the aspartic acid residue peptide bond. Of the 11 caspases, Caspase-3 is considered the main terminal cleavage enzyme in the apoptosis process [28]. Furthermore, Caspase-3 is responsible for the cleavage of the DNA repair enzyme PARP, which is another hallmark of apoptosis [12]. Therefore, our findings that allicin reduces the cleavage of Caspase-3 and PARP are consistent with a role for allicin in preventing apoptosis. Furthermore, Bax is a member of the Bcl-2 family that regulates apoptosis by controlling mitochondrial membrane channels. Bax was the first pro-apoptotic member of this family that was identified, and its expression is increased by a variety of well-characterized apoptotic agents, including H_2_O_2_ [13]. Therefore, the ability of allicin to reduce Bax activation also supports the idea that allicin protects HUVECs from apoptosis caused by H_2_O_2_.

We also demonstrated that allicin effectively reduces levels of MDA, a biomarker of oxidative stress, while simultaneously increasing the activity of SOD, an antioxidant enzyme. MDA levels indirectly reflect the severity of attack in cells by free radicals, and SOD activity levels indirectly reflect the capability of scavenging oxygen free radicals [14, 15]. Therefore, these findings suggest that allicin protects HUVECs by preventing oxidative stress. In addition to increasing antioxidant activity, allicin may be involved in the scavenging of oxygen free radicals, prevention of lipid peroxidation, and stabilization of the cell membrane.

Our results further show that H_2_O_2_ dramatically decreases nitric oxide (NO) levels in HUVEC culture medium, while allicin leads to increased NO. NO is an endogenous vascular relaxing factor that is produced in endothelial cells. It serves as a ubiquitin signaling molecule and regulates angiostasis in blood vessels and apoptosis in many cells [16]. H_2_O_2_ also up-regulates the expression of cell adhesion molecules. Activation of neutrophils induces the formation of non-ion-dependent NOS, and consumes a large amount of L-Arg. H_2_O_2_ also prompts an increase in calcium, which generates a large amount of O^2−^ to directly inactivate NO by activating the xanthine/xanthine oxidase system [29]. Therefore, the increased release of NO by allicin may serve to reverse the effects of H_2_O_2_ and protect cells through its antioxidant activity. H_2_O_2_ may also decrease NO release through its effects on the expression of eNOS, an enzyme that activates NO production [17]. We have shown by both reverse transcription PCR and real-time quantitative PCR that allicin reverses this decrease in eNOS mRNA expression, which suggests an additional mechanism that may regulate its ability to increase the release of NO and decrease the apoptosis rate. These results demonstrate that allicin protects HUVECs from apoptosis and elucidate a pathway by which protection is mediated via the reduction in oxidative stress.

## Conclusion

Allicin has powerful effects in protecting HUVECs from apoptosis. The protection occurs via a mechanism involving the reduction in oxidative stress, as measured by increased SOD and reduced MDA, NO and eNOS. There finding suggest that allicin functions as a powerful antioxidant. Further studies will be necessary to determine the direct effects of allicin on atherosclerosis.
